# Cell Surface Proteins in Hepatocellular Carcinoma: From Bench to Bedside

**DOI:** 10.3390/vaccines8010041

**Published:** 2020-01-24

**Authors:** Gabriel Siracusano, Maria Tagliamonte, Luigi Buonaguro, Lucia Lopalco

**Affiliations:** 1 Division of Immunology, Transplantation and Infectious Diseases, San Raffaele Scientific Institute, 20132 Milan, Italy; lopalco.lucia@hsr.it; 2 Cancer Immunoregulation Unit, Istituto Nazionale per lo Studio e la Cura dei Tumori IRCCS, “Fondazione Pascale”, 80131 Naples, Italy; m.tagliamonte@istitutotumori.na.it (M.T.); l.buonaguro@istitutotumori.na.it (L.B.)

**Keywords:** hepatocellular carcinoma, cell surface proteins, biomarker, therapeutic target, clinical trial

## Abstract

Cell surface proteins act as the go-between in carrying the information from the extracellular environment to the intracellular signaling proteins. However, these proteins are often deregulated in neoplastic diseases, including hepatocellular carcinoma. This review discusses several recent studies that have investigated the role of cell surface proteins in the occurrence and progression of HCC, highlighting the possibility to use them as biomarkers of the disease and/or targets for vaccines and therapeutics.

## 1. Introduction

Proteins at the cell surface mediate the response to intra- and extracellular stimuli, orchestrating the crosstalk between the cells and the surrounding environment. The cell surface proteome that is the profile of the proteins present on the plasma membrane at a specific time is currently under investigation. Their strategic localization and their involvement in signalling pathways regulating important biological processes raise the interest of the biomedical research since they represent a source of potential therapeutic targets and diagnostic/prognostic markers. In cancer, the aberrant expression of these proteins on neoplastic or neoplastic-associated cells, such as immune and endothelial cells, deeply affects normal biological functions and induce cancer proliferation, progression and recurrence. 

Hepatocellular carcinoma (HCC) is recognized as the second most common cause of cancer-related mortality and the most frequent diagnosed primary liver malignancy worldwide. Global HCC burden has risen to 841,080 new diagnosed cases and 781,631 deaths (GLOBOCAN database 2018), and the incidence is expected to increase in the next years [1,2]. The aggressiveness, the propensity to metastasize both intra- and extra-hepatic, and the frequent postoperative recurrence are the main characteristics of HCC. The late diagnosis caused by the paucity of specific symptoms and the limited spectrum of effective therapies are often responsible for the poor prognosis, making HCC a public health and economic concern.

The majority of HCC cases are associated with liver cirrhosis derived from Hepatitis B virus (HBV) or Hepatitis C virus (HCV) infections, chronic and autoimmune hepatitis. Alcohol abuse, non−alcoholic fatty liver disease (NAFLD), non−alcoholic steatohepatitis (NASH), aflatoxin B1 exposure, diabetes mellitus, obesity, and tobacco use are additional risk factors contributing to HCC development [2]. The multiplicity of risk factors associated with HCC, together with different pathogenesis, clinical course and prognosis contribute to the high heterogeneity of the disease, complicating the diagnosis and the treatment [3].

In the last decade, several progresses have been made in improving the prevention, the diagnosis and the treatment of HCC. Avoidance of the exposure to the risk factors potentially reduce the incidence of HCC. The implementation of vaccination programs against HBV significantly reduced the burden of HBV-related HCC [4]. In addition, the administration of antiviral agents blocking HBV and HCV chronic infections impaired the progression of the disease and probably HCC development [5,6,7]. Traditionally, surgical resection and liver transplantation are the treatments of choice indicated for early-stage HCC, resulting in a ~5-year survival expectancy. Transarterial chemoembolization (TACE) is usually recommended at intermediate stage, with variable 2- to 5-year survival rates, whereas at more advanced stage, only systemic therapies based on sorafenib and regorafenib administration are effective in improving the outcome of the patients. However, in this case, the overall survival is ~1 year [2]. However, chemotherapy is not effective for the emergence of drug resistance genes in some patients [8]. Importantly, novel immunotherapeutic interventions, especially those based on immune checkpoint inhibitors, have been entered in clinical trials showing promising results [9]. 

Nevertheless, the immunosuppressive microenvironment that characterizes the liver and the lack of tumor-associated antigens specific to HCC limit the efficacy of these treatments. Therefore, the current needs in the HCC research field rely on: i) the identification of new biomarkers for the early detection of HCC; ii) the discovery of new therapeutic targets to develop precision medicine approaches according to the subtypes of the disease, focusing on the characteristics of the individual patient. To those aims, the proteomic landscape of HCC is currently under investigation to identify differentially expressed proteins or neo-antigens specific for HCC that might promote the design of innovative clinical interventions to improve the management of HCC patients. 

The goal of this review is to provide an overview of the most relevant cell surface-bound proteins known to have a role in HCC tumorigenesis and progression, and their current or potential implication in the diagnosis, prevention and treatment of HCC. 

## 2. The Cell Surface Proteome of HCC

Integrated multi-omic approaches allowed the characterization of HCC tissues, along with in vitro models of HCC to dissect the molecular mechanisms of the disease. Several alterations of the cell surface proteome were attributed to both HCC cells and HCC microenvironment-associated cells (i.e., fibroblast, endothelial, and immune cells) and extracellular matrix (ECM) that play a pivotal role in supporting cancer proliferation, growth and invasion. 

**Figure 1:**
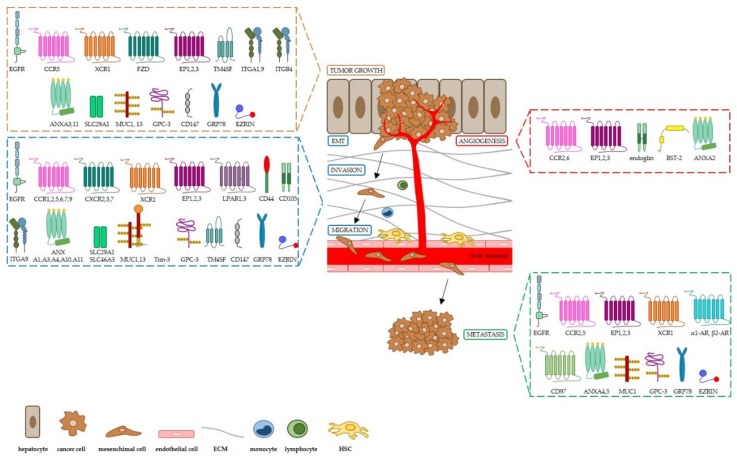
Schematic representation of cell surface receptors involved in hepatocarcinogenesis and HCC progression. EMT: epithelial–mesenchymal transition; ECM: extracellular matrix; HSC: hepatic stellate cells.

We overviewed selected dysregulated proteins and proteins related to altered signaling pathways with respect to their impact on HCC, as illustrated in Figure 1, and we grouped them according to their structural and biological function in receptors, cell adhesion molecules, transporters, mucins, glycosylphosphatidylinositol-anchored (GPI)-anchored proteins, and other cell surface-bound proteins. For each mentioned protein, we highlighted the molecular mechanisms known to have a role in hepatocarcinogenesis and tumor progression (i.e., tumor growth, angiogenesis, invasion, epithelial–mesenchimal transition (EMT), migration and metastasis) as well as their clinical relevance as biomarkers and/or target candidates for more effective therapies and vaccination strategies against the tumor. We apologize in advance for not reviewing other relevant studies in the field, due to space limitations. Finally, we underlined the completed and ongoing clinical trials targeting the mentioned cell surface proteins we focused on, summarized in Table 1.

## 3. Receptors

### 3.1. Tyrosine Kinase Receptor Family (TRK)

#### Epidermal Growth Factor Receptor (EGFR)

The epidermal growth factor receptor (EGFR) is a single chain transmembrane glycoprotein belonging to the tyrosine kinase receptor family (TRK), frequently expressed in epithelial tumors. Ito et al. found that EGFR was expressed in 68% of the HCC analyzed. The study highlighted a key role for EGFR in HCC progression since its expression correlated with high proliferation rate, advanced tumor stage, the presence of intrahepatic metastasis and poor disease-free survival [10]. Recently, López-Luque et al. described that EGFR is frequently downregulated in HCC patients, and the concomitant upregulation of TGF-β has prognostic value [11]. Several EGFR inhibitors have entered the clinical practice. Song et al. first reported the use of nimotuzumab, a humanized anti-EGFR monoclonal antibody (mAb) that blocks cancer cell proliferation, invasion, and metastasis, to treat an 87-year-old HCC patient, resulting in complete disease remission. This clinical case suggested that this mAb might be a promising alternative for HCC therapy, especially for those patients unable to respond to chemotherapy and surgery [12].

### 3.2. G-Protein-Coupled Receptors (GPCR)

G-protein-coupled receptors (GPCR) are a superfamily of seven transmembrane proteins acting as signal transducers that activate different downstream signaling pathways. They are divided into 6 classes: class A (rhodopsin-like), class B (secretin receptor family), class C (metabotropic glutamate/pheromone), class D (fungal mating pheromone receptors), class E (cyclic adenosine monophosphate (cAMP) receptors), and class F (frizzled/smoothened).

Several evidences revealed that aberrant expression of GPCR, especially those belonging to the class A and class F, had a crucial role in HCC tumorigenesis and progression, as extensively reviewed by Peng et al., and Chan and Lo [13,14].

#### 3.2.1. Chemokine Receptors

Chemokine receptors expressed on HCC cells regulate proliferation, migration, invasion and apoptosis of HCC cells after binding to their cognate ligands. Many evidences showed that these receptors trigger the activation of the phosphatidylinositol-4,5-bisphosphate 3-kinase (PI3K)/protein kinase B (Akt) signaling pathway, thus resulting in HCC occurrence and development.

CC chemokine receptor (CCR) 1 is highly expressed in human HCC tissues and the C-C motif chemokine ligand (CCL) CCL15/CCR1 axis has an important role in HCC cell migration and invasion through the matrix metalloproteases (MMP) MMP−2 and MMP-9-dependent extracellular matrix degradation [15]. Moreover, it was recently found that CCL15 induced the recruitment of CCR1^+^ CD14+ monocytes to HCC invasive margin; in turn, monocytes suppressed the anti-tumor immune response and promoted tumor metastasis. Therefore, blocking the CCL15/CCR1 axis might represent a promising therapeutic approach to reduce HCC growth and metastasis in vivo [16].

CCR2 and its ligand CCL2 are expressed by both tumoral and non-tumoral cells within HCC tissues [17], and the chemoattracting function of CCL2 was well established. High levels of CCL2 in tumoral and peritumoral tissues associated with poor patient survival. The CCR2 antagonist RDC018 inhibited HCC growth and metastasis and suppressed murine liver tumor growth via activating T cell antitumor immune response [18]. Bartneck et al. reported that CCR2^+^ tumor associated−macrophages 1 (TAM1) with pro-inflammatory and pro-angiogenic properties accumulated at HCC margin. Pharmacological inhibition of CCL2 impaired the CCR2^+^ TAM1 recruitment, thus suppressing angiogenesis and HCC progression [19]. The accumulation of macrophages and hepatic stellate cells was reduced in CCR2-deficient mice, thus affecting neovascularization [20]. Another study showed that CCL2 induced the migration of myeloid suppressor cells (MSCs) to tumor site, inducing tumor immune evasion [21]. Zhao et al. showed that the CCL2/CCR2 axis was involved in the recruitment of the myeloid cell subset CD11b/Gr1^mid^, responsible for the occurrence of colorectal cancer liver metastasis; moreover, the inhibition of the CCL2/CCR2 axis significantly affected tumor burden [22]. Additional evidences of the CCR2 involvement in promoting metastasis come from the study of Zhuang et al. showing that CCL2 promoted HCC invasion and EMT via the Hedgehog signaling activation [23]. By contrast, CCL2 induced the Th1 and CD8+ T cells and natural killer (NK) cells recruitment into the tumor microenvironment (TME), determining cancer cell death, thus contributing to prolonged patient survival [24]. 

CCR5/Rantes axis induced the recruitment of macrophages into the inflammation sites of the liver, playing a crucial role in the inflammation-induced tumorigenesis [17,25]. Of note, treatment with Maraviroc, the CCR5 antagonist, reduced mortality, liver fibrosis, and tumorigenesis in a mouse model of HCC [26]. A phase II clinical trial utilizes BMS-813160, a CCR2/5-inhibitor, in combination with nivolumab to examine the possibility to gain a significant anti-tumoral immune response and improvement in long term survival rates of HCC patients.

CCR6 plays a role in tumor progression and invasion. The CCR6/CCL20 axis, in fact, regulated IL-17-producing Th17 cells that accumulated in the TME, promoting angiogenesis [27]. Moreover, the axis mediated the migration of circulating Tregs in the TME, thus resulting in cancer progression and poor prognosis in patients [28]. Qiu et al. found that the cell surface nucleolin expressed in HCC tissues participated to the CCR6 signaling pathway, leading to adhesion, migration, and invasion of HCC cells. Therefore, nucleolin could be used as a biomarker of poor prognosis and as a therapeutic target in the clinical practice [29]. 

High expression of CCR7 was related to the processes of both intrahepatic and lymphatic invasion of HCC cells [30]. Interestingly, Chen et al. showed that the accumulation of CCL21, the cognate ligand of CCR7, created an anti-tumor environment due to the increase of both the number of T cells and dendritic cells and the levels of IL-12 and IFN-γ at tumor site. Therefore, they suggested that CCL21 is a promising tool for HCC immunotherapy [31]. A recent study reported that the HCC progression and the poor survival of patients may be associated with high CCR7 expression. The same authors highlighted a novel mechanism by which CCR7 up-regulation inhibition promoted EMT via histone deacetylase (HDAC) [32]. CCR7 is one of the immune biomarkers whose percentage changes are analyzed in an observational study of HCC patients who undergo lobar TheraSphere radioembolization.

CCR9 is notably up-regulated in HCC tissues, and this was related to the presence of several tumor nodes, high Edmondson-Steiner grade and vascular invasion. CCR9 promoted tumor proliferation by increasing the number of HCC cells at S phase, increasing the expression of the cell cycle regulators p21 and p27and concomitantly reducing cyclin D1 levels [33].

High levels of C-X-C chemokine receptor (CXCR) 2 correlated with progression and poor prognosis in human HCC. The CXCL5/CXCR2 axis induced EMT and promoted the migration and invasion of HCC cells by activating the PI3K/Akt/GSK-3β/Snail and PI3K/Akt/ERK1/2 signaling pathways [34]. In addition, CXCL5 induced the infiltration of peri-tumoral neutrophils, promoting tumor angiogenesis and growth [35]. Li et al. found that the CXCR2/CXCL1 axis correlated with the infiltration of CD15+ neutrophil into both intra- and peri-tumoral sites. The axis represented an independent prognostic factor in HCC as well [36].

CXCL9 binding to CXCR3 activated extracellular signal-regulated kinases 1 and 2 (ERK1/2) signaling, with consequent increased MMP2 and MMP9 expression that, in turn, promoted the invasion and metastasis activity of the CD133+ cancer stem cells [37]. An additional study showed that the up-regulation of the chemokine interferon-γ inducible protein 10 kD (CXCL10)/CXCR3 signaling determined HCC recurrence after transplantation, by inducing the mobilization and the recruitment of Tregs at liver graft injury [38]. A phase I clinical trial analyzes the safety of sitagliptin treatment, a dipeptidylpeptidase IV inhibitor, as a strategy for protecting CXCL10 activity as a means to enhance tumor regression.

Several studies have highlighted a crucial role of CXCR4 during HCC progression. The CXCL12-mediated perinuclear translocation of CXCR4 in Huh7/Hep3B cells increased the invasiveness of Huh7 cells. In addition, CXCR4 expression in HCC tissues strikingly correlated with progressed local tumors, lymphatic metastasis, distant dissemination, and decreased 3-year survival rate [39,40]. Bertran et al. observed that CXCR4 localization at tumor border and perivascular space might contribute to tumor dissemination [41]. The crosstalk between CXCL12/CXCR4 axis and different molecules was observed. The crosstalk with the transforming growth factor-β (TGF-β) increased cancer cell migration [41], whereas the CXCL12/CXCR4-mediated MMP10 expression via the ERK1/2 signaling pathway contributed to the HCC progression and metastasis [42].

Monnier et al. found marked CXCR7 expression on endothelial cells under hypoxic and acidic pH conditions, typical of TME [43]. Increased CXCR7 expression was found in HCC tissues, where it played a critical role in proliferation, migration, invasion and angiogenesis of HCC cells [44,45,46].

High expression of CXCL16 and CXCR6 promoted HCC invasiveness and a pro-tumor inflammatory environment caused by the recruitment of neutrophils. This event associated with poor patient outcome; therefore, the inhibition of this pathway may improve the prognosis after HCC treatment [47]. Oral administration of vancomycin altered gut commensal bacteria in mice, increasing CXCL16 expression of liver sinusoidal endothelial cells, thus recruiting hepatic CXCR6+ NKT cells that exert anti-tumor effect [48]. A phase II trial examines whether oral vancomycin therapy affects the relative CXCR6 gene expression levels in the liver in paired pre- and on-treatment biopsy samples from hepatic lesions in patients with unresectable fibrolamellar HCC, a rare liver cancer.

Interestingly, high levels of CX3CL1/CX3CR1 in HCC tissues correlated with fewer intra- and extrahepatic recurrences, low PCNA labeling index (PCNALI), leading to a better prognosis regarding disease-free and overall survival of patients [49]. Therefore, CX3CR1 could be considered a prognostic marker for patients with HCC and a good target for immunotherapies to prevent HCC.

Yanru et al. demonstrated that X-C motif chemokine receptor 1 (XCR1) played a dual role in HCC, impairing tumor growth and tumorigenesis, via MAPK and PI3K/Akt signaling pathways, whereas it promoted EMT that induced metastasis [50].

#### 3.2.2. E-Prostanoid Receptors

E-prostanoid receptors EP1, EP2, EP3, and EP4 are a class of GPCR that binds to prostaglandin E2 (PGE2). They have a role in HCC tumorigenesis and progression, modulating the growth, adhesion, invasion, metastasis and angiogenesis of tumor cells [51]. EP1 overexpression in HCC cells promoted cell invasion via EMT [52]. In addition, EP1 induced β1-integrin expression which, in turns, activated the protein kinase C (PKC)/ nuclear factor kappa B (NF-κB) signaling pathway, thus resulting in HCC migration [53]. Breinig et al. demonstrated that the PGE2 protumorigenic effects are transmitted via EP1 and EP3 receptors in HCC cells, since EP1 and EP3-receptor-antagonism decreased cell viability and induced apoptosis in HCC cells. Therefore, the authors suggested that targeting EP1 and EP3-dependent signaling might be a chemotherapeutic strategy against HCC. [54].

PGE2 binding to EP2 mediated the up-regulation of the Snail protein through the EP2/Src/EGFR/Akt/mTOR pathway in Huh-7 cells, promoting HCC cell invasion and migration [55]. Moreover, PGE2 upregulated c-Myc expression through the EP4R/GS/AC/cAMP/PKA/CREB signaling pathway, thus promoting cell growth and invasion in HCC cells [51].

#### 3.2.3. Lysophosphatidic Acid Receptors

There are six identified lysophosphatidic acid (LPA) receptors (LPAR1-6) normally involved in the regulation of proliferation, motility and migration. Sokolow et al. reported changes in LPAR1, 3 and 6 mRNA and protein expression in HCC and, in particular, a significant increase of LPAR6 in HCC compared to normal and non-tumor livers. In vitro studies on hepatoma cell lines have shown that LPA treatment induced the growth and increased the motility of HuH7 cells in a dose-dependent manner, whereas it moderately increased the proliferation of HepG2 cells, without affecting their motility [56]. Mazzocca et al. demonstrated that LPA mediated the recruitment of peritumoral tissue fibroblasts that transdifferentiated into myofibroblasts, enhancing proliferation, migration, and invasion of HCC cells. Therefore, the authors suggested that LPA inhibition could effectively impair tumor progression [57]. Park et al. found that MMP-9 was a downstream effector of LPAR1 in HCC tissues. LPAR1 up-regulation induced MMP-9 by a mechanism involving the phosphatidylinositol 3-kinase (PI3K) and the mitogen activated protein kinase (MAPK) p38 signaling pathways, with subsequent increase of the HCC cell invasiveness [58]. Zuckerman et al. observed high levels LPAR1 and LPAR3 expression in the microenvironment between the tumor and non-tumor liver as well as in SKHep1 cells. In addition, the authors found a subset of LPAR3+ cancer stem cells in the same region that might mediate HCC invasion and expansion via LPA-LPAR3 signaling [59].

#### 3.2.4. Adrenergic Receptors

Adrenergic receptors are a class of GPCR, which are the targets of catecholamines to regulate sympathetic neurotransmission and other biological processes. α1-AR was downregulated in HCC tissues, leading to alterations of the cancer metabolism of carbohydrates and wasting syndrome in patients [60]. Activated α1-AR and β2-AR have a role in promoting tumor metastasis by a mechanism involving the release of EGF-like ligands by the MMP-7 [61]. In addition, high levels of β2-AR associated with poor prognosis after resection. Wu et al. showed that β2-AR was a negative regulator of autophagy, leading to hypoxia-inducible factor-1α stabilization, reprogramming the glucose metabolism of HCC cells, and the resistance to sorafenib; therefore antagonist of this receptor could represent novel therapeutic tools for HCC and chemoresistance [62].

#### 3.2.5. Frizzled Receptors (FZD)

Frizzled (FZD) proteins are a class of GPCR composed by ten members (FZD1-10) involved in the canonical Wnt/β-catenin signaling pathway, frequently activated in HCC. Besides mutations of specific genes, deregulation of key mediators of this pathway, including FZD receptors, was reported to affect Wnt activation, as suggested by the increased expression of β-catenin target genes in HCC as a consequence of the up-regulation of FZD7 [63].

An extensive transcriptomic analysis revealed that the up-regulation of FZD3/6/7 and Wnt3/4/5, and the concomitant downregulation of the antagonists of the Wnt pathway sFRP1/5 are common features in the 95% HCC and 68% peritumors. The high expression of the three FZD was associated with the activation of downstream signaling pathways involving not only the canonical β-catenin, but the noncanonical PKC and c-Jun N-terminal kinases (JNK) pathways as well. In addition, FZD7 dysregulation frequently associated with HBV-related HCC, whereas dysregulation of FZD3/6 was homogeneously distributed among HBV, HCV, and non-B, non-C (NBNC) hepatitis-related HCCs [64].

Several studies improved the knowledge on the functional role of FZD7 in HCC. Deregulation of FZD7 generally occurs at the early steps of the hepatocarcinogenesis [63,65]. A study highlighted that FZD7 is the direct transcriptional target of Sox 9, the sex-determining region Y (SRY)-related high-mobility-group box transcription factors member, that drives the FZD7-mediated activation of the Wnt signaling pathway, conferring stem cell-like features to HCC cells [66]. A recent study showed that FZD7 is also a target of miR-504, which exerts a suppressive role in HCC through inhibition of the Wnt signaling and, consequently, tumor progression. Therefore, the mir-507/FZD7/Wnt signaling axis might be a potential target for antitumor therapies [67]. In this line, pharmacological inhibitors or soluble FZD7 peptide aimed at inhibiting the function of FZD7 are thought to act as antitumor tools on HCC both in vitro and in vivo [68,69]. 

FZD2 transcripts were found to be overexpressed in late-stage clinical cases of HCC and correlated with poor patient survival [70]. An additional study showed that FZD2 up-regulation induced EMT and enhanced cell migration and invasiveness, and related to poor recurrence-free survival [71]. An in vitro study reported that the Let7b-mediated downregulation of FZD4 inhibited the Wnt/β-catenin signaling, reducing the ratios of liver cancer stem cells and impairing the proliferation, invasion, and migration of liver cancer cells [72]. In a mouse model of HCC, FZD6 upregulation contributed to tumor progression, by a mechanism regulated by miR-194 [73]. FZD9 has reported to have an important role in HCC tumorigenesis, inducing cell proliferation and motility of HCC cell lines [74]. Capurro et al. reported that FZD4/7/8 interacted with the heparin sulfate chains of glypican-3, triggering the formation of a signaling complex between Wnts and FZD at the cell membrane [75].

#### 3.2.6. Adhesion Receptors

Cluster of differentiation 97 (CD97) is a member is a member of the adhesion subfamily of GPCR. Yin et al. have been recently described human CD97-high and CD97-low HCC samples. No correlation with age, gender, hepatitis B surface antigen, hepatitis C virus, cirrhosis, alpha-fetoprotein, and Edmondson grade were reported. On the other hand, CD97 overexpression correlated to poorer relapse-free survival, increased vascular invasion, intrahepatic and distant metastasis, and tumor size. The authors suggested that the stimulatory role of CD97 in HCC metastasis relied on an integrated regulatory interaction between CD97 and GRK6: In absence of GRK6, CD97 up-regulation promoted MMP2 and MMP9 secretion, thereby facilitating metastasis. Of note, overexpression of CD97 in HCC cells accelerated lung metastasis in vivo [76].

#### 3.2.7. G protein Receptor Kinases (GRKs)

Wei et al. reported that the G protein coupled receptor kinase 2 (GRK2) negatively regulated the TRK family member insulin-like growth factor-1 receptor (IGF-1R) signaling pathway in HepG2 cells. Indeed, GRK2 knockdown increased IGF-1R expression and phosphorylation, without improving cell growth, and induced a small cell cycle arrest at G2/M phase by enhancing the expression of cyclin A, B1, and E as well. [77]. Ma et al found that GRK2, by downregulating the early growth response-1 (EGR1) expression, inhibited the IGF1-induced HCC cell growth and migration, suggesting that GRK2 might represent a therapeutic approach in HCC treatment [78].

### 3.3. Cluster of Differentiation 44 (CD44)

The cluster of differentiation 44 (CD44) is a transmembrane glycoprotein receptor for several ligands, including ialuronic acid, osteopontin, collagen, fibronectin, and a co-receptor for EGFR and c-Met. 

CD44 is expressed by the myeloid cells in the liver under healthy conditions, namely Kupffer cells and lymphocytes [79]. However, during cancer progression, CD44 expression increased in HCC progenitor cells (HcPCs) [80,81]. Moreover, together with epithelial cell adhesion molecule (EpCAM), CD133, CD90, CD24, and CD13, CD44 was also characterized as a cancer stem cell (CSC) marker [82]. Dhar et al. have recently gave insight into the involvement of CD44 in HCC. They found that CD44 protected hepatocytes from DNA damage by inhibiting the apoptotic response and the cell cycle arrest mediated by the tumor suppressor protein p53, thus resulting in carcinogenesis [81].

As a consequence of alternative mRNA splicing, the standard CD44 isoform (CD44s) and the variant CD44 isoforms (CD44v) exist [83]. Asai et al. found that CD44s was essential for maintaining cancer stemness of the hepatoma cell line HuH7 through a mechanisms involving NOTCH3 and regulated the oxidative stress response as well [84]. 

Gao et al. reported that CD44 is essential in maintaining the mesenchymal phenotype, since CD44 knockdown reversed EMT, inhibiting the invasion and metastasis of HCC both in vitro and in vivo, partially due to the inhibition of ERK/Snail signaling pathway [85]. Interestingly, Yang et al. have recently reported that high levels of cholesterol induced CD44 translocation into lipid rafts, and attenuated CD44-ezrin binding, which is essential for migration of cancer cells and the formation of metastasis. The authors suggested that therapeutic approaches aimed at retaining CD44 might increase the survival of HCC patients [86]. 

### 3.4. Endoglin (CD105)

Endoglin is a transmembrane glycoprotein expressed by activated endothelial cells acting as a co-receptor for the TGF-β. Endoglin is preferentially expressed on activated liver sinusoidal endothelial cells with high angiogenic, migration, and anti-apoptotic properties. Kasprzak and Adamek extensively reviewed the role of endoglin in HCC angiogenesis [87]. TRC105, a chimeric IgG1 anti-CD105 monoclonal antibody, was tested in a phase I/II clinical trial to evaluate whether the combination therapy is more effective than sorafenib alone to arrest tumor growth and reduce tumor size for HCC patients who did not respond to other treatments.

### 3.5. Receptor for Adenovirus and Coxsackievirus (CAR)

The receptor for adenovirus and coxsackievirus (CAR), a type 1 transmembrane component of the tight junction complex, is involved in the binding of group B coxsachievirus and a number of adenovirus subtypes [88]. Evidences coming from the analysis of the expression in several malignancies revealed that CAR is differentially regulated depending on the type of tumor. High levels of CAR were detected in HCC at advanced stages of the disease [89].

### 3.6. Bone Marrow Stromal Cell Antigen 2 (BST2)

The bone marrow stromal cell antigen 2 (also referred as BST2, tetherin, CD317, or HM1.24 antigen) is a type II transmembrane glycoprotein that tethers budding enveloped virus on the cell membrane for their subsequent endocytosis and degradation at the lysosomal compartment [90,91]. BST2 inhibited the release of DENV virions from Huh7 cells and limited viral cell-to-cell transmission. BST2 overexpression was reported in several tumors. Li et al. uncovered the anti-apoptotic function of BST2 in serum-starved Hep-G2 cells. The authors suggested that BST2 can be a useful target for combined antiangiogenic cancer therapies, since cancer cells can experience nutrient deprivation during progression and/or treatments that disrupt vascularization [92].

## 4. Cell Adhesion Molecules

### Integrins

Integrins are integral membrane glycoproteins composed of noncovalently associated α- and β-subunits, resulting in 24 known heterodimers. Integrins have two main physiological roles: They act as cell surface receptors and mediates cell adhesion to the extracellular matrix (ECM), triggering signaling pathways that regulates proliferation, differentiation and motility [93]. 

High levels of β1 integrin (ITGB1) were detected in HCC tissues and multicellular spheroids of HCC cells [94]. Several studies reported that ITGB1 overexpression associated with tumor progression and drug resistance [95,96]. Tian et al. highlighted the role of ITGB1 in preventing HCC cells proliferation inhibition and apoptosis induced by chemotherapy drugs through the activation of the focal adhesion kinase (FAK)/Akt signaling pathway [94]. Of note, ITGB1-dependent activation of the FAK pathway was promoted by collagen I deposition in NASH, thus resulting in HCC proliferation [97]. Zhang et al. first reported that integrin α9β1 (ITGA9) decreased in HCC patients and negatively correlated with HCC proliferation, migration, and invasion through the FAK/Src-Rho GTPase signaling pathway [98]. MINT1526A (RG-7594), a humanized monoclonal antibody that blocks the interaction of α5β1 integrin with its ECM ligands, showed good tolerability and preliminary evidences of efficacy in combination with bevacizumab in a phase I clinical trial [99].

Integrin β4 (ITGB4) overexpression was detected in both HCC tissues and hepatoma cell lines. In vitro and in vivo evidences showed that ITGB4 promoted HCC proliferation, EMT, invasion, and metastasis by a mechanism involving the transcription factor Slug through AKT/Sox2-Nanog pathway [100].

## 5. Transporters

### 5.1. Annexins (ANXs)

Annexins are a family of Ca^2+^-dependent phospholipid-binding proteins, located on the surface of most eukaryotic cells. Among the five groups of annexins (A–E), human annexins belong to group A including 12 members (ANXA1–A11 and ANXA13). Each member exerts different functions involved in vesicle trafficking, cell signaling, ion transport, cell division, and apoptosis. Structurally, annexins are characterized by two domains: a conserved protein core of highly and tightly packed α-helix, containing the Ca^2+^ and the membrane binding sites, and an N-terminal domain specific for any member that confers the different properties [101]. 

Dysregulation of annexins expression was reported in a variety of cancers, including HCC. 

Yu et al. detected high expression levels of ANXA1 in poorly differentiated rather than well-differentiated or moderately differentiated HCC cell lines such as Mahlavu and SK-Hep-1 cells, suggesting a role of ANXA1 in the invasive capacity of HCC cells that improved their metastatic potential [102]. 

A cDNA microarray analysis revealed that ANXA2 gene was up-regulated in the sinusoidal endothelium and in the malignant hepatocytes of HCC tissues, thus indicating that it might be a marker for angiogenesis in HCC [103]. Yan et al. found that in cancer-associated mesenchymal stem cells (MSC), ANXA2 was bound by the long noncoding RNA lncRNA-MUF, a novel critical player in the MSC-mediated HCC progression. This interaction triggered the activation of the Wnt/β-catenin signaling pathway, accelerating the EMT process [104]. Moreover, the serum of HCC patients at early stage of the disease contained higher ANXA2 concentrations compared to those of healthy subjects, further suggesting that ANXA2 played an important role in HCC progression [105]. Shaker et al. investigated the clinical utility of ANXA2 serum levels as a novel diagnostic marker of HCC in Egyptian patients, suggesting that ANXA2 might be a good biomarker for the early detection of HCC for the higher sensitivity, specificity, and positive and negative predictive values compared to that of alpha-fetoprotein (AFP) [106].

Pan et al. reported that ANXA3 was up-regulated at both mRNA and protein level in human HCC tissues compared to the adjacent non-tumoral tissues. They found that ANXA3 overexpression correlated with more aggressive HCC characteristics, including the presence of multiple tumor lesions, larger tumors, and advanced tumor stage and, consequently, poor prognosis of HCC patients [107]. Tong et al. also demonstrated that ANXA3 was the most significant up-regulated protein in the liver subset of CD133+ cancer stem cells and both the endogenous and the secreted proteins had a role in promoting HCC aggressiveness and stem cell-like properties [108]. The same group has recently reported that the enrichment in ANXA3 conferred resistance to sorafenib therapy to HCC cells by a mechanism partially involving the concomitant induction of autophagy and suppression of PKCδ/p38-dependent apoptosis, thereby promoting cell survival. Interestingly, they found that an anti-ANXA3 mAb blocked cell proliferation inducing cell death in vitro, and enhanced the sensitivity of HCC patient-derived organoids to sorafenib treatment. Therefore, this study highlighted that ANXA3 is a useful target to treat the resistance to sorafenib therapy [109]. 

A large cohort-study carried out by Chen et al. showed that ANXA4 expression was significantly up-regulated in HCC tumor with early recurrence/metastasis and correlated with both the clinicopathologic characteristics and the poor overall survival of HCC patients. The overexpression of ANXA4 in vitro increased HCC cell migration and invasion via the EMT regulation, supporting a potential role for ANXA4 in HCC progression [110].

High levels of ANXA5 correlated with the up-regulation of the two isoforms of the adapter molecule crk (CRKI/II) and the Ras-related C3 botulinum toxin substrate 1 (RAC1) in HCC tissues, potentially increasing the clinical progression and the lymphatic metastasis of patients. The inhibition of ANXA5 deeply affected the expression of key proteins involved in both the integrin (CRKI/II, DOCK180, RAC1) and mitogen-activated protein kinase kinase (MEK)-ERK signaling pathways (p-MEK, p-ERK, c-Myc, MMP-9), indicating that ANXA5 is a potential target for HCC diagnosis and treatment [111].

Unlike the previous mentioned members of the annexins family, both ANXA6 and ANXA10 expressions were found to be downregulated in HCC. The role of ANXA6 in liver physiology is well documented [112]. Meier et al. demonstrated that ANXA6 protein levels, but not mRNAs, were lower in HCC tissues compared to adjacent non-tumoral tissues; however, the role of this protein as potential prognostic marker has not been further investigated yet [113].

Downregulation of ANXA10 associated with malignant phenotype of liver cells, and correlated with vascular invasion, early recurrence and poor prognosis of HCC patients, suggesting that ANXA10 might be a potential tumor suppressor gene [114]. 

Liu et al. have recently shown that the long non-coding RNA AGAP2-AS1, by sponging miR-16-5p, up-regulated ANXA11, the direct downstream target of miR-16-5p, resulting in Akt signaling pathway activation. The authors suggested that the AGAP2-AS1/miR-16-5p/ANXA11/AKT axis had a critical role in HCC progression since it promoted proliferation, migration and invasion and inhibited apoptosis both in vitro and in vivo, and it might be a target for HCC therapies [115]. 

### 5.2. Solute Carrier Transporters (SLCs)

In humans there are 395 solute carriers, divided in 52 families based on their sequence similarities, involved in the transport of small molecules across the plasma membrane, such as inorganic ions, amino acids, lipids, neurotransmitters, and drugs [116]. A number of studies demonstrated that aberrant expression of these proteins associated with multi-drug resistance in HCC. 

Low SLC29A1 expression correlated with high recurrence rates of HCC after surgery and poor prognosis for patients. Low SLC29A1 expression enhanced tumor cell proliferation and invasion both in vitro and in vivo, and reduced drug sensitivity through changing cell adhesion status, induction of EMT, and nuclear factor-kappaB (NF-κB) pathway activation [117].

SLC2A2 (GLUT2) expression was higher in HCC tissues compared to that of SLC2A family members. Increased expression of SLC2A2 correlated with advanced clinical stage and independently associated with overall survival in patients with HCC, suggesting that SLC2A2 might be considered a prognostic factor for HCC [118].

Low levels of the gene solute carrier family 46 (sodium phosphate), member 3 (SLC46A3) were detected in the 83.2% of human HCC tissues compared to non-tumor adjacent tissues by Zhang et al. [119]. Downregulation of SLC46A3 blocked EMT in HCC, impairing the occurrence of metastasis, thus suggesting that this protein might be used in therapies aimed at targeting EMT. SLC46A3 also affected sorafenib sensitivity of HCC by a mechanism that has not been investigated yet. In addition, they found that SLC46A3 was a powerful prediction marker for prognosis.

## 6. Mucins

Mucins are a family of 22 secreted and membrane-bound large glycosylated proteins (MUC1-22) composed of 80% carbohydrate and 20% core proteins. Mucins contain tandem repeat structures rich in Proline, Threonine and Serine, which constitute the PTS domain. The threonine and serine residues in the PTS domain are heavily O-glycosylation through N-acetylgalactosamine O-linkages (GalNAcO-linkages) [120]. Aberrant expression of mucins was reported in several neoplastic gastrointestinal organs. 

Cao et al. demonstrated that mucin 1 (MUC1) was highly expressed in HCC and can be considered as an indicator of HCC prognosis [121]. MUC1 localized at the apical surface of epithelial cells, and was overexpressed not only in HCC but in hepatoma cells as well, playing a relevant role in tumorigenesis [122]. Wang et al. gave further insights on the role of MUC1, showing that it induced the MMP-9-mediated HCC cell migration and invasion via the c-Jun N-terminal kinase (JNK)-dependent phosphorylation of Smad2 [123]. Lin et al. detected higher MUC1 expression levels in HCC patients with cardiac metastasis than in those with primary HCC alone, and the first had a poorer prognosis compared to the latter [124]. Targeting MUC1 by antibody-derived chimeric antigen receptor (CAR) T or CAR-pNK therapies is the purpose of phase I/II trials in patients with MUC1 positive relapsed or refractory solid tumors, including HCC. In addition, the adenoviral MUC1 vaccine ETBX-061, in combination with innate high-affinity Natural Killer (haNK) cell therapy and yeast-based vaccines, is used in a phase Ib/II trial to study the induction of T-cell responses in patients with advanced, unresectable, and untransplantable HCC.

Dai et al. found that mucin 13 (MUC13) upregulation, detected in 44% HCC tissues, promoted the transition G1/S via the Wnt signaling, inducing hepatocarcinogenesis and tumor progression. High MUC13 expression associated with tumor encapsulation, tumor size, venous invasion, tumor stage, and poor outcome of patients [125].

Mucin15 (MUC15) is frequently reduced in HCC tissues and negatively correlated with high serum AFP levels, vascular invasion, lack of encapsulation, poor differentiation, hepatitis B e antigen positivity, early and higher recurrence, and shorter survival times after resection. MUC15 interaction with EGFR reduced EGFR dimerization, increasing its endocytosis with consequent degradation, thereby reducing the PI3K-Akt-dependent HCC progression and metastasis [126]. 

Lin et al. reported a role of the T cell immunoglobulin and mucin-domain containing-3 (Tim-3) in regulating EMT occurrence and further metastasis of HCC in vitro [127]. As recently extensively reviewed by Liu et al., Tim-3 is a new discovered immune checkpoint molecule playing a relevant role in the development of HCC [128]. Tim-3 is a potential marker of prognosis and can be used be used to evaluate both the prognosis and therapeutic effects in HCC. Preclinical experiments showed that targeting Tim-CD147 had anti-tumor effects; therefore it might be a new target for cancer therapy [129]. TSR-022, an anti-TIM-3 antibody, was used in combination with the anti-PD-1 antibody TSR-042 in a phase II trial to examine whether they may arise an anti-tumoral immune response that blocks cancer proliferation in patients with locally advanced or metastatic liver cancer. 

## 7. Glycosylphosphatidylinositol-Anchored Proteins

### Glypican-3

Glypican-3 (GPC−3) is a heparansulfate (HS) proteoglycan attached to the cell surface by a glycosylphosphatidylinositol (GPI)-containing anchor [130]. It is normally expressed in placenta and several embryonic tissues, adult ovary, mammary gland, mesothelium, lung, and kidney. GPC−3 expression is low or absent in normal adult liver tissues, cirrhosis or benign liver lesions. By contrast, several studies have demonstrated that GPC−3 mRNAs and protein expression levels are high in hepatic cancer cells [131,132,133]. A recent meta-analysis revealed that GPC−3 overexpression strictly associated with poor overall survival (OS) and disease-free survival (DFS) in HCC patients. The same study highlighted a correlation with HBV infection, vascular invasion, late stage and high tumor grade as well [134]. GPC−3 promoted the growth of hepatoma cells by activating the canonical Wnt signaling pathway [135,136,137]. In addition, Ruan et al. showed GPC−3 involvement in HCC proliferation and metastasis both in vitro and in vivo [138]. An additional study reported that GPC3−mediated activation of the ERK signaling pathway induces EMT in cancer cells, thus resulting in HCC progression and metastasis [139].

As GPC−3 is specifically expressed in hepatic tumor cells, it is considered a good target for peptide vaccines against HCC. Monoclonal antibodies against the core of the GPC−3 protein were obtained and tested in preclinical and clinical studies [140,141,142]. Of note, phase I clinical trial of codrituzumab, a humanized monoclonal antibody recognizing an epitope in the C-terminal region of GPC−3, has already demonstrated good tolerance in patients with advanced HCC [141,143], but no efficacy in phase II study [144]. None of those antibodies was reported to affect the Wnt/β-catenin signaling pathway. Conversely, the human monoclonal antibody recognizing the HS chains of GPC−3 developed by Gao et al. was shown to impair cell proliferation in vitro and HCC xenograft tumors in mice by blocking the Wnt/β-catenin signaling pathway [145]. 

Innovative GPC-3 targeting-therapies, in particular those based on GPC-3 chimeric antigen receptor T (CAR-T) cells showed promising results in killing GPC-3 positive HCC cells [146]. A phase I clinical study demonstrated high safety profile and efficacy of GPC-3 CAR-T cells in patients with relapsed or refractory HCC [147] and several clinical trial based on GPC-3 CAR-T cells alone or in combination with lymphodepleting therapy are ongoing in patients with advanced HCC. Interestingly, the combination of GPC-3 CAR-T cells with sorafenib or anti-PD-1, demonstrated effective clinical potential in mouse models of HCC [148,149].

## 8. Other Cell Surface-Associated Proteins

### 8.1. Human Tetraspanin Transmembrane 4 Superfamily (TM4SF)

The human tetraspanin transmembrane 4 superfamily member 4 (TM4SF), also named il-TMP, was originally identified at intestinal epithelium and liver level [150]. Li and colleagues showed that TM4SF mRNA and protein levels are overexpressed in the 80% of HCC tissues compared to the adjacent non-tumor tissues. Whereas no correlation between TM4SF expression and age, gender or tumor size was identified, immunohistochemical analysis revealed that the higher expression of TM4SF4 occurred in well and moderately differentiated tumors and at early stages as well [151]. Therefore, this protein is a good candidate as early diagnostic and prognostic marker of HCC. An anti-TM4SF5 monoclonal antibody showed antitumor activity in vitro, as demonstrated by the reduced cell proliferation and motility, and the enhanced adhesion. This mAb demonstrated therapeutic efficacy in a mouse model of HCC as well [152].

### 8.2. Cluster of Differentiation 147 (CD147)

CD147 (also referred as basigin, EMMPRIN, or HAb 18G) is a transmembrane glycoprotein belonging to the immunoglobulin superfamily. CD147 expression associated with carcinogenesis, EMT, and chemoresistance in HCC [153,154,155,156]. More recently, the meta-analysis carried out by Peng et al. highlighted that high CD147 expression may be related to the survival, TNM stage, and venous invasion in patients with HCC [157].

N-glycosylation modifications regulate the biological functions of CD147. β1,6-N-acetylglucosamine (β1,6-GlcNAc) glycans linked to CD147 increased MMP expression and enhanced the interaction of CD147 with integrin β1, promoting HCC metastasis. No association with tumor stage, cirrhosis, differentiation, lymph node metastasis, HBsAg, and serum AFP levels was reported [158]. During EMT, theGnT-V-mediated link of β1,6-N-acetylglucosamine (β1,6-GlcNAc) glycans to CD147 increased. This event enhanced the MMP expression and the interaction of CD147 with integrin β1, leading to the downstream stimulation of the Rac/Ras/Raf/ERK and PI3K/Akt pathways, that enhanced the invasive and metastatic potential of HCC cells. Therefore, affecting the N-glycosylation modification of CD147 might be a novel approach for the development of therapeutic strategies targeting metastasis [158]. A study on the safety and clinical activity of CD147-targeted CAR-T cell therapy by hepatic artery infusions for very advanced HCC is ongoing. In a multicenter phase IV clinical study, patients with unresectable HCC were treated with licartin, an antibody with high affinity for HAb18G/CD147, in combination with TACE, showing promising tolerability and efficacy.

### 8.3. Glucose-Regulated Protein 78 (GRP78)

The 78-KDa glucose-regulated protein (GRP78), also known as BiP and heat shock 70 kDa protein 5 (HSPA5), is localized at the endoplasmic reticulum in healthy conditions, but it is expressed at high levels on the cell surface of many types of tumors, including HCC. Cell surface GRP78 association with activated α2-macroglobin stimulated the invasion and metastasis of HCC, by a c-Src-dependent mechanism [159]. In addition, GRP78 increased HCC proliferation in vitro and in vivo by increasing the ubiquitin-like protein human leukocyte antigen-F adjacent transcript 10 (FAT10) expression [160].

### 8.4. Ezrin

Ezrin is a member of the ERM (ezrin–radixin–moesin) cytoskeleton-associated protein family. It is involved in maintaining cell shape and polarity and participates in several cellular activities including signaling, growth, and differentiation. The essential role of ezrin in regulating HCC proliferation, migration, and invasiveness was shown in vitro [161]. In HCC tissues, high levels of ezrin expression associated with advanced Tumor, Nodes, Metastases (TNM) stage, poor Edmondson’s histological grade, macroscopic portal vein invasion, tumor recurrence and extrahepatic recurrence. Kang et al. suggested that the molecular mechanism by which ezrin promote HCC progression and metastasis might involve the signaling triggered by c-Met, whereas no association with the previous mentioned CD44 or E-cadherin was found [162]. Dedifferentiation and invasion of HBV-infected HCC cells were attributed to ezrin overexpression, which independently associated with tumors with smaller size, cirrhotic liver background, poor differentiation, and vascular invasion in HBV-HCC patients [163]. Ezrin gene expression was reduced by the treatment with arsenic trioxide, which inhibit HCC invasion and metastasis by blocking the RhoC signaling pathway [164].

## 9. Conclusions

Epidemiological data point out the importance of HCC as a major public health and economic concern. HCC diagnosis is difficult to assess and the current treatments are not effective to ensure a positive clinical outcome in patients. Therefore, the identification of both clinical prognostic and/or diagnostic markers and targets to develop more effective therapies are still a clinical unmet need.

To our knowledge, there is not yet a review that summarizes some of the most relevant cell surface proteins involved in HCC occurrence, progression and recurrence and their potential or current targeting for vaccine and/or therapeutic-based approaches. 

Cell surface proteins become attractive candidates to respond to the challenges in the field of cancer, including HCC, for their strategic localization and their involvement in signaling pathways often altered in cancer. Those proteins may be useful for the diagnosis of HCC as novel biomarkers and for the treatment of HCC as therapeutic targets. Several proteins involved in those processes were identified by proteomics, implementing the genomic information, therefore improving the spectrum of putative candidates. Importantly, several cell surface proteins have been already selected as targets for immunotherapy and entered onto clinical trials, showing promising results.

The importance of the investigation rely on the fact that one of the major obstacles in HCC treatment is the lack of tumor-associated antigens highly specific for HCC able to elicit a tumor immune response, therefore enabling the design of more potent intervention strategies, e.g. therapeutic cancer vaccines.

However, the growing interest for the cell surface protein profiling has to face with some challenges including the detection of low abundant and insoluble proteins, reversible post-translational modifications (i.e., phosphorylation, glycosylation), and the need for huge amount of starting material that limit the amount of recovered proteins. Several approaches for improving the enrichment and the solubilization of plasma membrane-associated proteins have been developed, along with cutting-edge technologies aimed at improving the comprehensive analysis of many type of cancers. However, the in depth characterization of the HCC cell surface proteome is still needed. Novel and fine-tuning techniques should be developed and combination strategies should provide more successful methods of investigation. Importantly, a tighter collaboration between clinicians and researchers should be envisaged in order to facilitate the access to both clinical specimens and clinical-pathological characteristics of the tumor. This aspect is relevant to patient stratification for precision medicine. Indeed, in recent years, the personalized medicine approach aimed at identifying cell surface proteins that are unique for the individual patient in order to overcome either the limit of the heterogeneity that characterizes certain types of disease, especially HCC, and the side effects on chemotherapy. This approach results in the development of more effective therapies according to the subtype of the disease, optimized per individual tumor or patient. 

## Figures and Tables

**Table 1:** Summary of the clinical trials registered in ClinicalTrial.gov. N/A: not applicable.

Target	Studies	Phase	ClinicalTrial.gov Identifier
EGFR	21	I/II; I; II; I/II;	NCT02273362; NCT01219543; NCT00752063; NCT03499626;
		II; II; II; I; II;	NCT03203837; NCT00532441; NCT00071994; NCT00047346;
		I; I; II; II;	NCT00365391; NCT03319459; NCT03829436; NCT00107536;
		N/A; I; I;	NCT00033462; NCT03176485; NCT04106167; NCT03841110;
		II; N/A; N/A;	NCT02465060; NCT03042520; NCT01043523; NCT03170960;
		I/II; I	NCT03993873
CCR2/5	1	II	NCT04123379
CCR7	1	NA	NCT03203837
CXCL10	1	I	NCT02650427
CXCR6	1	II	NCT04025567
CD105	3	I/II; II; I/II	NCT02560779; NCT01375569; NCT01306058
ANXA2	1	NA	NCT02541149
MUC1	3	I/II; I/II; I/II	NCT02587689; NCT02839954; NCT03563170
Tim-3		II	NCT03680508
GPC-3	16	NA; I; I/II; I;	NCT03146234; NCT02905188; NCT03084380; NCT03884751; NCT03980288; NCT04121273; NCT02723942; NCT02395250; NCT04093648; NCT03198546; NCT02715362; NCT03130712; NCT03086564; NCT03175705; NCT03302403; NCT02959151
		I; I; I/II; I;	
		I; I; I/II;	
		I/II; I/II; I;	
		N/A; I/II	
CD147	2	I; IV	NCT03993743; NCT00829465

## References

[1] Fact Sheets by Cancer. http://globocan.iarc.fr/Pages/fact_sheets_cancer.aspx.

[2] Forner A., Reig M., Bruix J. (2018). Hepatocellular carcinoma. Lancet.

[3] Liang Q., Shen X., Sun G. (2018). Precision Medicine: Update on Diagnosis and Therapeutic Strategies of Hepatocellular Carcinoma. Curr. Med. Chem..

[4] Chang M.-H., You S.-L., Chen C.-J., Liu C.-J., Lai M.-W., Wu T.-C., Wu S.-F., Lee C.-M., Yang S.-S., Chu H.-C. (2016). Long-term Effects of Hepatitis B Immunization of Infants in Preventing Liver Cancer. Gastroenterology.

[5] Papatheodoridis G.V., Chan H.L.-Y., Hansen B.E., Janssen H.L.A., Lampertico P. (2015). Risk of hepatocellular carcinoma in chronic hepatitis B: Assessment and modification with current antiviral therapy. J. Hepatol..

[6] Singal A.K., Singh A., Jaganmohan S., Guturu P., Mummadi R., Kuo Y.-F., Sood G.K. (2010). Antiviral therapy reduces risk of hepatocellular carcinoma in patients with hepatitis C virus-related cirrhosis. Clin. Gastroenterol. Hepatol..

[7] Morgan R.L., Baack B., Smith B.D., Yartel A., Pitasi M., Falck-Ytter Y. (2013). Eradication of hepatitis C virus infection and the development of hepatocellular carcinoma: A meta-analysis of observational studies. Ann. Intern. Med..

[8] Le Grazie M., Biagini M.R., Tarocchi M., Polvani S., Galli A. (2017). Chemotherapy for hepatocellular carcinoma: The present and the future. World J. Hepatol..

[9] Prieto J., Melero I., Sangro B. (2015). Immunological landscape and immunotherapy of hepatocellular carcinoma. Nat. Rev. Gastroenterol. Hepatol..

[10] Ito Y., Takeda T., Sakon M., Tsujimoto M., Higashiyama S., Noda K., Miyoshi E., Monden M., Matsuura N. (2001). Expression and clinical significance of erb-B receptor family in hepatocellular carcinoma. Br. J. Cancer.

[11] López-Luque J., Bertran E., Crosas-Molist E., Maiques O., Malfettone A., Caja L., Serrano T., Ramos E., Sanz-Moreno V., Fabregat I. (2019). Downregulation of Epidermal Growth Factor Receptor in hepatocellular carcinoma facilitates Transforming Growth Factor-β-induced epithelial to amoeboid transition. Cancer Lett..

[12] Song P., Yang J., Li X., Huang H., Guo X., Zhou G., Xu X., Cai Y., Zhu M., Wang P. (2017). Hepatocellular carcinoma treated with anti-epidermal growth factor receptor antibody nimotuzumab. Medicine.

[13] Peng W.-T., Sun W.-Y., Li X.-R., Sun J.-C., Du J.-J., Wei W. (2018). Emerging Roles of G Protein-Coupled Receptors in Hepatocellular Carcinoma. Int. J. Mol. Sci..

[14] Chan K.K.-S., Lo R.C.-L. (2018). Deregulation of Frizzled Receptors in Hepatocellular Carcinoma. Int. J. Mol. Sci..

[15] Li Y., Wu J., Zhang P. (2016). CCL15/CCR1 axis is involved in hepatocellular carcinoma cells migration and invasion. Tumour Biol..

[16] Liu L.-Z., Zhang Z., Zheng B.-H., Shi Y., Duan M., Ma L.-J., Wang Z.-C., Dong L.-Q., Dong P.-P., Shi J.-Y. (2019). CCL15 Recruits Suppressive Monocytes to Facilitate Immune Escape and Disease Progression in Hepatocellular Carcinoma. Hepatology.

[17] Fantuzzi L., Tagliamonte M., Gauzzi M.C., Lopalco L. (2019). Dual CCR5/CCR2 targeting: Opportunities for the cure of complex disorders. Cell. Mol. Life Sci..

[18] Li X., Yao W., Yuan Y., Chen P., Li B., Li J., Chu R., Song H., Xie D., Jiang X. (2017). Targeting of tumour-infiltrating macrophages via CCL2/CCR2 signalling as a therapeutic strategy against hepatocellular carcinoma. Gut.

[19] Bartneck M., Schrammen P.L., Möckel D., Govaere O., Liepelt A., Krenkel O., Ergen C., McCain M.V., Eulberg D., Luedde T. (2018). The CCR2^+^ Macrophage Subset Promotes Pathogenic Angiogenesis for Tumor Vascularization in Fibrotic Livers. Cell. Mol. Gastroenterol. Hepatol..

[20] Yang X., Lu P., Ishida Y., Kuziel W.A., Fujii C., Mukaida N. (2006). Attenuated liver tumor formation in the absence of CCR2 with a concomitant reduction in the accumulation of hepatic stellate cells, macrophages and neovascularization. Int. J. Cancer.

[21] Huang B., Lei Z., Zhao J., Gong W., Liu J., Chen Z., Liu Y., Li D., Yuan Y., Zhang G.-M. (2007). CCL2/CCR2 pathway mediates recruitment of myeloid suppressor cells to cancers. Cancer Lett..

[22] Zhao L., Lim S.Y., Gordon-Weeks A.N., Tapmeier T.T., Im J.H., Cao Y., Beech J., Allen D., Smart S., Muschel R.J. (2013). Recruitment of a myeloid cell subset (CD11b/Gr1 mid) via CCL2/CCR2 promotes the development of colorectal cancer liver metastasis. Hepatology.

[23] Zhuang H., Cao G., Kou C., Liu T. (2018). CCL2/CCR2 axis induces hepatocellular carcinoma invasion and epithelial-mesenchymal transition in vitro through activation of the Hedgehog pathway. Oncol. Rep..

[24] Chew V., Chen J., Lee D., Loh E., Lee J., Lim K.H., Weber A., Slankamenac K., Poon R.T.P., Yang H. (2012). Chemokine-driven lymphocyte infiltration: An early intratumoural event determining long-term survival in resectable hepatocellular carcinoma. Gut.

[25] Barashi N., Weiss I.D., Wald O., Wald H., Beider K., Abraham M., Klein S., Goldenberg D., Axelrod J., Pikarsky E. (2013). Inflammation-induced hepatocellular carcinoma is dependent on CCR5 in mice. Hepatology.

[26] Ochoa-Callejero L., Pérez-Martínez L., Rubio-Mediavilla S., Oteo J.A., Martínez A., Blanco J.R. (2013). Maraviroc, a CCR5 antagonist, prevents development of hepatocellular carcinoma in a mouse model. PLoS ONE.

[27] Zhang J.-P., Yan J., Xu J., Pang X.-H., Chen M.-S., Li L., Wu C., Li S.-P., Zheng L. (2009). Increased intratumoral IL-17-producing cells correlate with poor survival in hepatocellular carcinoma patients. J. Hepatol..

[28] Chen K.-J., Lin S.-Z., Zhou L., Xie H.-Y., Zhou W.-H., Taki-Eldin A., Zheng S.-S. (2011). Selective recruitment of regulatory T cell through CCR6-CCL20 in hepatocellular carcinoma fosters tumor progression and predicts poor prognosis. PLoS ONE.

[29] Qiu W., Wang G., Sun X., Ye J., Wei F., Shi X., Lv G. (2015). The involvement of cell surface nucleolin in the initiation of CCR6 signaling in human hepatocellular carcinoma. Med. Oncol..

[30] Schimanski C.C., Bahre R., Gockel I., Junginger T., Simiantonaki N., Biesterfeld S., Achenbach T., Wehler T., Galle P.R., Moehler M. (2006). Chemokine receptor CCR7 enhances intrahepatic and lymphatic dissemination of human hepatocellular cancer. Oncol. Rep..

[31] Chen L., Zhou S., Qin J., Hu H., Ma H., Liu B., Wang X., Ma J., Ye S., Zhong C. (2013). Combination of SLC administration and Tregs depletion is an attractive strategy for targeting hepatocellular carcinoma. Mol. Cancer.

[32] Yang L., Chang Y., Cao P. (2018). CCR7 preservation via histone deacetylase inhibition promotes epithelial-mesenchymal transition of hepatocellular carcinoma cells. Exp. Cell Res..

[33] Zhang Z., Qin C., Wu Y., Su Z., Xian G., Hu B. (2014). CCR9 as a prognostic marker and therapeutic target in hepatocellular carcinoma. Oncol. Rep..

[34] Liu Z., Yang L., Xu J., Zhang X., Wang B. (2011). Enhanced expression and clinical significance of chemokine receptor CXCR2 in hepatocellular carcinoma. J. Surg. Res..

[35] Kuang D.-M., Zhao Q., Wu Y., Peng C., Wang J., Xu Z., Yin X.-Y., Zheng L. (2011). Peritumoral neutrophils link inflammatory response to disease progression by fostering angiogenesis in hepatocellular carcinoma. J. Hepatol..

[36] Li L., Xu L., Yan J., Zhen Z.-J., Ji Y., Liu C.-Q., Lau W.Y., Zheng L., Xu J. (2015). CXCR2-CXCL1 axis is correlated with neutrophil infiltration and predicts a poor prognosis in hepatocellular carcinoma. J. Exp. Clin. Cancer Res..

[37] Ding Q., Xia Y., Ding S., Lu P., Sun L., Liu M. (2016). An alternatively spliced variant of CXCR3 mediates the metastasis of CD133^+^ liver cancer cells induced by CXCL9. Oncotarget.

[38] Li C.X., Ling C.C., Shao Y., Xu A., Li X.C., Ng K.T.-P., Liu X.B., Ma Y.Y., Qi X., Liu H. (2016). CXCL10/CXCR3 signaling mobilized-regulatory T cells promote liver tumor recurrence after transplantation. J. Hepatol..

[39] Schimanski C.C., Bahre R., Gockel I., Müller A., Frerichs K., Hörner V., Teufel A., Simiantonaki N., Biesterfeld S., Wehler T. (2006). Dissemination of hepatocellular carcinoma is mediated via chemokine receptor CXCR4. Br. J. Cancer.

[40] Xiang Z., Zeng Z., Tang Z., Fan J., Sun H., Wu W., Tan Y. (2010). [Nuclear accumulation of CXCR4 and overexpressions of VEGF-C and CK19 are associated with a higher risk of lymph node metastasis in hepatocellular carcinoma]. Chin. J. Oncol..

[41] Bertran E., Crosas-Molist E., Sancho P., Caja L., Lopez-Luque J., Navarro E., Egea G., Lastra R., Serrano T., Ramos E. (2013). Overactivation of the TGF-β pathway confers a mesenchymal-like phenotype and CXCR4-dependent migratory properties to liver tumor cells. Hepatology.

[42] García-Irigoyen O., Latasa M.U., Carotti S., Uriarte I., Elizalde M., Urtasun R., Vespasiani-Gentilucci U., Morini S., Benito P., Ladero J.M. (2015). Matrix metalloproteinase 10 contributes to hepatocarcinogenesis in a novel crosstalk with the stromal derived factor 1/C-X-C chemokine receptor 4 axis. Hepatology.

[43] Monnier J., Boissan M., L’Helgoualc’h A., Lacombe M.-L., Turlin B., Zucman-Rossi J., Théret N., Piquet-Pellorce C., Samson M. (2012). CXCR7 is up-regulated in human and murine hepatocellular carcinoma and is specifically expressed by endothelial cells. Eur. J. Cancer.

[44] Xue T.-C., Chen R.-X., Han D., Chen J., Xue Q., Gao D.-M., Sun R.-X., Tang Z.-Y., Ye S.-L. (2012). Down-regulation of CXCR7 inhibits the growth and lung metastasis of human hepatocellular carcinoma cells with highly metastatic potential. Exp. Ther. Med..

[45] Lin Q., Peng S., Yang Y. (2018). Inhibition of CD9 expression reduces the metastatic capacity of human hepatocellular carcinoma cell line MHCC97-H. Int. J. Oncol..

[46] Zheng K., Li H.-Y., Su X.-L., Wang X.-Y., Tian T., Li F., Ren G.-S. (2010). Chemokine receptor CXCR7 regulates the invasion, angiogenesis and tumor growth of human hepatocellular carcinoma cells. J. Exp. Clin. Cancer Res..

[47] Gao Q., Zhao Y.-J., Wang X.-Y., Qiu S.-J., Shi Y.-H., Sun J., Yi Y., Shi J.-Y., Shi G.-M., Ding Z.-B. (2012). CXCR6 upregulation contributes to a proinflammatory tumor microenvironment that drives metastasis and poor patient outcomes in hepatocellular carcinoma. Cancer Res..

[48] Ma C., Han M., Heinrich B., Fu Q., Zhang Q., Sandhu M., Agdashian D., Terabe M., Berzofsky J.A., Fako V. (2018). Gut microbiome-mediated bile acid metabolism regulates liver cancer via NKT cells. Science.

[49] Matsubara T., Ono T., Yamanoi A., Tachibana M., Nagasue N. (2007). Fractalkine-CX3CR1 axis regulates tumor cell cycle and deteriorates prognosis after radical resection for hepatocellular carcinoma. J. Surg. Oncol..

[50] Yanru W., Zhenyu B., Zhengchuan N., Qi Q., Chunmin L., Weiqiang Y. (2018). Transcriptomic analyses of chemokines reveal that down-regulation of XCR1 is associated with advanced hepatocellular carcinoma. Biochem. Biophys. Res. Commun..

[51] Xia S., Ma J., Bai X., Zhang H., Cheng S., Zhang M., Zhang L., Du M., Wang Y., Li H. (2014). Prostaglandin E2 promotes the cell growth and invasive ability of hepatocellular carcinoma cells by upregulating c-Myc expression via EP4 receptor and the PKA signaling pathway. Oncol. Rep..

[52] Zhang H., Cheng S., Zhang M., Ma X., Zhang L., Wang Y., Rong R., Ma J., Xia S., Du M. (2014). Prostaglandin E2 promotes hepatocellular carcinoma cell invasion through upregulation of YB-1 protein expression. Int. J. Oncol..

[53] Bai X., Wang J., Guo Y., Pan J., Yang Q., Zhang M., Li H., Zhang L., Ma J., Shi F. (2014). Prostaglandin E2 stimulates β1-integrin expression in hepatocellular carcinoma through the EP1 receptor/PKC/NF-κB pathway. Sci. Rep..

[54] Breinig M., Rieker R., Eiteneuer E., Wertenbruch T., Haugg A.M., Helmke B.M., Schirmacher P., Kern M.A. (2008). Differential expression of E-prostanoid receptors in human hepatocellular carcinoma. Int. J. Cancer.

[55] Cheng S.-Y., Zhang H., Zhang M., Xia S.-K., Bai X.-M., Zhang L., Ma J., Rong R., Wang Y.-P., Du M.-Z. (2014). Prostaglandin E₂ receptor EP2 mediates Snail expression in hepatocellular carcinoma cells. Oncol. Rep..

[56] Sokolov E., Eheim A.L., Ahrens W.A., Walling T.L., Swet J.H., McMillan M.T., Simo K.A., Thompson K.J., Sindram D., McKillop I.H. (2013). Lysophosphatidic acid receptor expression and function in human hepatocellular carcinoma. J. Surg. Res..

[57] Mazzocca A., Dituri F., Lupo L., Quaranta M., Antonaci S., Giannelli G. (2011). Tumor-secreted lysophostatidic acid accelerates hepatocellular carcinoma progression by promoting differentiation of peritumoral fibroblasts in myofibroblasts. Hepatology.

[58] Park S.Y., Jeong K.J., Panupinthu N., Yu S., Lee J., Han J.W., Kim J.M., Lee J.-S., Kang J., Park C.G. (2011). Lysophosphatidic acid augments human hepatocellular carcinoma cell invasion through LPA1 receptor and MMP-9 expression. Oncogene.

[59] Zuckerman V., Sokolov E., Swet J.H., Ahrens W.A., Showlater V., Iannitti D.A., Mckillop I.H. (2016). Expression and function of lysophosphatidic acid receptors (LPARs) 1 and 3 in human hepatic cancer progenitor cells. Oncotarget.

[60] Kassahun W.T., Günl B., Jonas S., Ungemach F.R., Abraham G. (2011). Altered liver α1-adrenoceptor density and phospholipase C activity in the human hepatocellular carcinoma. Eur. J. Pharmacol..

[61] Li J., Yang X.-M., Wang Y.-H., Feng M.-X., Liu X.-J., Zhang Y.-L., Huang S., Wu Z., Xue F., Qin W.-X. (2014). Monoamine oxidase A suppresses hepatocellular carcinoma metastasis by inhibiting the adrenergic system and its transactivation of EGFR signaling. J. Hepatol..

[62] Wu F.-Q., Fang T., Yu L.-X., Lv G.-S., Lv H.-W., Liang D., Li T., Wang C.-Z., Tan Y.-X., Ding J. (2016). ADRB2 signaling promotes HCC progression and sorafenib resistance by inhibiting autophagic degradation of HIF1α. J. Hepatol..

[63] Merle P., de la Monte S., Kim M., Herrmann M., Tanaka S., Von Dem Bussche A., Kew M.C., Trepo C., Wands J.R. (2004). Functional consequences of frizzled-7 receptor overexpression in human hepatocellular carcinoma. Gastroenterology.

[64] Bengochea A., de Souza M.M., Lefrançois L., Le Roux E., Galy O., Chemin I., Kim M., Wands J.R., Trepo C., Hainaut P. (2008). Common dysregulation of Wnt/Frizzled receptor elements in human hepatocellular carcinoma. Br. J. Cancer.

[65] Merle P., Kim M., Herrmann M., Gupte A., Lefrançois L., Califano S., Trépo C., Tanaka S., Vitvitski L., de la Monte S. (2005). Oncogenic role of the frizzled-7/beta-catenin pathway in hepatocellular carcinoma. J. Hepatol..

[66] Leung C.O.-N., Mak W.-N., Kai A.K.-L., Chan K.-S., Lee T.K.-W., Ng I.O.-L., Lo R.C.-L. (2016). Sox9 confers stemness properties in hepatocellular carcinoma through Frizzled-7 mediated Wnt/β-catenin signaling. Oncotarget.

[67] Quan H., Li B., Yang J. (2018). MicroRNA-504 functions as a tumor suppressor in hepatocellular carcinoma through inhibiting Frizzled-7-mediated-Wnt/β-catenin signaling. Biomed. Pharmacother..

[68] Nambotin S.B., Lefrancois L., Sainsily X., Berthillon P., Kim M., Wands J.R., Chevallier M., Jalinot P., Scoazec J.-Y., Trepo C. (2011). Pharmacological inhibition of Frizzled-7 displays anti-tumor properties in hepatocellular carcinoma. J. Hepatol..

[69] Wei W., Chua M.-S., Grepper S., So S.K. (2011). Soluble Frizzled-7 receptor inhibits Wnt signaling and sensitizes hepatocellular carcinoma cells towards doxorubicin. Mol. Cancer.

[70] Gujral T.S., Chan M., Peshkin L., Sorger P.K., Kirschner M.W., MacBeath G. (2014). A noncanonical Frizzled2 pathway regulates epithelial-mesenchymal transition and metastasis. Cell.

[71] Asano T., Yamada S., Fuchs B.C., Takami H., Hayashi M., Sugimoto H., Fujii T., Tanabe K.K., Kodera Y. (2017). Clinical implication of Frizzled 2 expression and its association with epithelial-to-mesenchymal transition in hepatocellular carcinoma. Int. J. Oncol..

[72] Cai H., Chen Y., Yang X., Ma S., Wang Q., Zhang Y., Niu X., Ding G., Yuan Y. (2017). Let7b modulates the Wnt/β-catenin pathway in liver cancer cells via downregulated Frizzled4. Tumour Biol..

[73] Krützfeldt J., Rösch N., Hausser J., Manoharan M., Zavolan M., Stoffel M. (2012). MicroRNA-194 is a target of transcription factor 1 (Tcf1, HNF1α) in adult liver and controls expression of frizzled-6. Hepatology.

[74] Fujimoto T., Tomizawa M., Yokosuka O. (2009). SiRNA of frizzled-9 suppresses proliferation and motility of hepatoma cells. Int. J. Oncol..

[75] Capurro M., Martin T., Shi W., Filmus J. (2014). Glypican-3 binds to Frizzled and plays a direct role in the stimulation of canonical Wnt signaling. J. Cell Sci..

[76] Yin Y., Xu X., Tang J., Zhang W., Zhangyuan G., Ji J., Deng L., Lu S., Zhuo H., Sun B. (2018). CD97 Promotes Tumor Aggressiveness Through the Traditional G Protein-Coupled Receptor-Mediated Signaling in Hepatocellular Carcinoma. Hepatology.

[77] Wei Z., Hurtt R., Gu T., Bodzin A.S., Koch W.J., Doria C. (2013). GRK2 negatively regulates IGF-1R signaling pathway and cyclins’ expression in HepG2 cells. J. Cell. Physiol..

[78] Ma Y., Han C.-C., Huang Q., Sun W.-Y., Wei W. (2016). GRK2 overexpression inhibits IGF1-induced proliferation and migration of human hepatocellular carcinoma cells by downregulating EGR1. Oncol. Rep..

[79] Flanagan B.F., Dalchau R., Allen A.K., Daar A.S., Fabre J.W. (1989). Chemical composition and tissue distribution of the human CDw44 glycoprotein. Immunology.

[80] He G., Dhar D., Nakagawa H., Font-Burgada J., Ogata H., Jiang Y., Shalapour S., Seki E., Yost S.E., Jepsen K. (2013). Identification of liver cancer progenitors whose malignant progression depends on autocrine IL-6 signaling. Cell.

[81] Dhar D., Antonucci L., Nakagawa H., Kim J.Y., Glitzner E., Caruso S., Shalapour S., Yang L., Valasek M.A., Lee S. (2018). Liver Cancer Initiation Requires p53 Inhibition by CD44-Enhanced Growth Factor Signaling. Cancer Cell.

[82] Yamashita T., Wang X.W. (2013). Cancer stem cells in the development of liver cancer. J. Clin. Investig..

[83] Ponta H., Sherman L., Herrlich P.A. (2003). CD44: From adhesion molecules to signalling regulators. Nat. Rev. Mol. Cell Biol..

[84] Asai R., Tsuchiya H., Amisaki M., Makimoto K., Takenaga A., Sakabe T., Hoi S., Koyama S., Shiota G. (2019). CD44 standard isoform is involved in maintenance of cancer stem cells of a hepatocellular carcinoma cell line. Cancer Med..

[85] Gao Y., Ruan B., Liu W., Wang J., Yang X., Zhang Z., Li X., Duan J., Zhang F., Ding R. (2015). Knockdown of CD44 inhibits the invasion and metastasis of hepatocellular carcinoma both in vitro and in vivo by reversing epithelial-mesenchymal transition. Oncotarget.

[86] Yang Z., Qin W., Chen Y., Yuan B., Song X., Wang B., Shen F., Fu J., Wang H. (2018). Cholesterol inhibits hepatocellular carcinoma invasion and metastasis by promoting CD44 localization in lipid rafts. Cancer Lett..

[87] Kasprzak A., Adamek A. (2018). Role of Endoglin (CD105) in the Progression of Hepatocellular Carcinoma and Anti-Angiogenic Therapy. Int. J. Mol. Sci..

[88] Cohen C.J., Shieh J.T., Pickles R.J., Okegawa T., Hsieh J.T., Bergelson J.M. (2001). The coxsackievirus and adenovirus receptor is a transmembrane component of the tight junction. Proc. Natl. Acad. Sci. USA.

[89] Reeh M., Bockhorn M., Görgens D., Vieth M., Hoffmann T., Simon R., Izbicki J.R., Sauter G., Schumacher U., Anders M. (2013). Presence of the coxsackievirus and adenovirus receptor (CAR) in human neoplasms: A multitumour array analysis. Br. J. Cancer.

[90] Neil S.J.D., Zang T., Bieniasz P.D. (2008). Tetherin inhibits retrovirus release and is antagonized by HIV-1 Vpu. Nature.

[91] Yang H., Wang J., Jia X., McNatt M.W., Zang T., Pan B., Meng W., Wang H.-W., Bieniasz P.D., Xiong Y. (2010). Structural insight into the mechanisms of enveloped virus tethering by tetherin. Proc. Natl. Acad. Sci. USA.

[92] Li X., Zhang G., Chen Q., Lin Y., Li J., Ruan Q., Chen Y., Yu G., Wan X. (2016). CD317 Promotes the survival of cancer cells through apoptosis-inducing factor. J. Exp. Clin. Cancer Res..

[93] Sökeland G., Schumacher U. (2019). The functional role of integrins during intra- and extravasation within the metastatic cascade. Mol. Cancer.

[94] Tian T., Li C.-L., Fu X., Wang S.-H., Lu J., Guo H., Yao Y., Nan K.-J., Yang Y.-J. (2018). β1 integrin-mediated multicellular resistance in hepatocellular carcinoma through activation of the FAK/Akt pathway. J. Int. Med. Res..

[95] Speicher T., Siegenthaler B., Bogorad R.L., Ruppert R., Petzold T., Padrissa-Altes S., Bachofner M., Anderson D.G., Koteliansky V., Fässler R. (2014). Knockdown and knockout of β1-integrin in hepatocytes impairs liver regeneration through inhibition of growth factor signalling. Nat. Commun..

[96] Jiang X., Wang J., Zhang K., Tang S., Ren C., Chen Y. (2015). The role of CD29-ILK-Akt signaling-mediated epithelial-mesenchymal transition of liver epithelial cells and chemoresistance and radioresistance in hepatocellular carcinoma cells. Med. Oncol..

[97] Zheng X., Liu W., Xiang J., Liu P., Ke M., Wang B., Wu R., Lv Y. (2017). Collagen I promotes hepatocellular carcinoma cell proliferation by regulating integrin β1/FAK signaling pathway in nonalcoholic fatty liver. Oncotarget.

[98] Zhang Y.-L., Xing X., Cai L.-B., Zhu L., Yang X.-M., Wang Y.-H., Yang Q., Nie H.-Z., Zhang Z.-G., Li J. (2018). Integrin α9 Suppresses Hepatocellular Carcinoma Metastasis by Rho GTPase Signaling. J. Immunol. Res..

[99] Weekes C.D., Rosen L.S., Capasso A., Wong K.M., Ye W., Anderson M., McCall B., Fredrickson J., Wakshull E., Eppler S. (2018). Phase I study of the anti-α5β1 monoclonal antibody MINT1526A with or without bevacizumab in patients with advanced solid tumors. Cancer Chemother. Pharmacol..

[100] Li X.-L., Liu L., Li D.-D., He Y.-P., Guo L.-H., Sun L.-P., Liu L.-N., Xu H.-X., Zhang X.-P. (2017). Integrin β4 promotes cell invasion and epithelial-mesenchymal transition through the modulation of Slug expression in hepatocellular carcinoma. Sci. Rep..

[101] Schloer S., Pajonczyk D., Rescher U. (2018). Annexins in Translational Research: Hidden Treasures to Be Found. Int. J. Mol. Sci..

[102] Wang R.-C., Huang C.-Y., Pan T.-L., Chen W.-Y., Ho C.-T., Liu T.-Z., Chang Y.-J. (2015). Proteomic Characterization of Annexin l (ANX1) and Heat Shock Protein 27 (HSP27) as Biomarkers for Invasive Hepatocellular Carcinoma Cells. PLoS ONE.

[103] Yu G.R., Kim S.H., Park S.H., Cui X.D., Xu D.Y., Yu H.C., Cho B.H., Yeom Y.I., Kim S.S., Kim S.B. (2007). Identification of molecular markers for the oncogenic differentiation of hepatocellular carcinoma. Exp. Mol. Med..

[104] Yan X., Zhang D., Wu W., Wu S., Qian J., Hao Y., Yan F., Zhu P., Wu J., Huang G. (2017). Mesenchymal Stem Cells Promote Hepatocarcinogenesis via lncRNA-MUF Interaction with ANXA2 and miR-34a. Cancer Res..

[105] Ji N.Y., Park M.-Y., Kang Y.H., Lee C.I., Kim D.G., Yeom Y.I., Jang Y.J., Myung P.-K., Kim J.W., Lee H.G. (2009). Evaluation of annexin II as a potential serum marker for hepatocellular carcinoma using a developed sandwich ELISA method. Int. J. Mol. Med..

[106] Shaker M.K., Abdel Fattah H.I., Sabbour G.S., Montasser I.F., Abdelhakam S.M., El Hadidy E., Yousry R., El Dorry A.K. (2017). Annexin A2 as a biomarker for hepatocellular carcinoma in Egyptian patients. World J. Hepatol..

[107] Pan Q.-Z., Pan K., Weng D.-S., Zhao J.-J., Zhang X.-F., Wang D.-D., Lv L., Jiang S.-S., Zheng H.-X., Xia J.-C. (2015). Annexin A3 promotes tumorigenesis and resistance to chemotherapy in hepatocellular carcinoma. Mol. Carcinog..

[108] Tong M., Fung T.-M., Luk S.T., Ng K.-Y., Lee T.K., Lin C.-H., Yam J.W., Chan K.W., Ng F., Zheng B.-J. (2015). ANXA3/JNK Signaling Promotes Self-Renewal and Tumor Growth, and Its Blockade Provides a Therapeutic Target for Hepatocellular Carcinoma. Stem Cell Rep..

[109] Tong M., Che N., Zhou L., Luk S.T., Kau P.W., Chai S., Ngan E.S., Lo C.-M., Man K., Ding J. (2018). Efficacy of annexin A3 blockade in sensitizing hepatocellular carcinoma to sorafenib and regorafenib. J. Hepatol..

[110] Chen W., Chen L., Cai Z., Liang D., Zhao B., Zeng Y., Liu X., Liu J. (2016). Overexpression of annexin A4 indicates poor prognosis and promotes tumor metastasis of hepatocellular carcinoma. Tumour Biol..

[111] Sun X., Liu S., Wang J., Wei B., Guo C., Chen C., Sun M.-Z. (2018). Annexin A5 regulates hepatocarcinoma malignancy via CRKI/II-DOCK180-RAC1 integrin and MEK-ERK pathways. Cell Death Dis..

[112] Enrich C., Rentero C., Grewal T. (2017). Annexin A6 in the liver: From the endocytic compartment to cellular physiology. Biochim. Biophys. Acta Mol. Cell Res..

[113] Meier E.M., Rein-Fischboeck L., Pohl R., Wanninger J., Hoy A.J., Grewal T., Eisinger K., Krautbauer S., Liebisch G., Weiss T.S. (2016). Annexin A6 protein is downregulated in human hepatocellular carcinoma. Mol. Cell. Biochem..

[114] Liu S.-H., Lin C.-Y., Peng S.-Y., Jeng Y.-M., Pan H.-W., Lai P.-L., Liu C.-L., Hsu H.-C. (2002). Down-regulation of annexin A10 in hepatocellular carcinoma is associated with vascular invasion, early recurrence, and poor prognosis in synergy with p53 mutation. Am. J. Pathol..

[115] Liu Z., Wang Y., Wang L., Yao B., Sun L., Liu R., Chen T., Niu Y., Tu K., Liu Q. (2019). Long non-coding RNA AGAP2-AS1, functioning as a competitive endogenous RNA, upregulates ANXA11 expression by sponging miR-16-5p and promotes proliferation and metastasis in hepatocellular carcinoma. J. Exp. Clin. Cancer Res..

[116] Lin L., Yee S.W., Kim R.B., Giacomini K.M. (2015). SLC transporters as therapeutic targets: Emerging opportunities. Nat. Rev. Drug Discov..

[117] Gao P.-T., Cheng J.-W., Gong Z.-J., Hu B., Sun Y.-F., Cao Y., Qiu S.-J., Zhou J., Fan J., Yang X.-R. (2017). Low SLC29A1 expression is associated with poor prognosis in patients with hepatocellular carcinoma. Am. J. Cancer Res..

[118] Kim Y.H., Jeong D.C., Pak K., Han M.-E., Kim J.-Y., Liangwen L., Kim H.J., Kim T.W., Kim T.H., Hyun D.W. (2017). SLC2A2 (GLUT2) as a novel prognostic factor for hepatocellular carcinoma. Oncotarget.

[119] Zhao Q., Zheng B., Meng S., Xu Y., Guo J., Chen L.-J., Xiao J., Zhang W., Tan Z.-R., Tang J. (2019). Increased expression of SLC46A3 to oppose the progression of hepatocellular carcinoma and its effect on sorafenib therapy. Biomed. Pharmacother..

[120] Dhanisha S.S., Guruvayoorappan C., Drishya S., Abeesh P. (2018). Mucins: Structural diversity, biosynthesis, its role in pathogenesis and as possible therapeutic targets. Crit. Rev. Oncol. Hematol..

[121] Cao Y., Karsten U., Otto G., Bannasch P. (1999). Expression of MUC1, Thomsen-Friedenreich antigen, Tn, sialosyl-Tn, and alpha2,6-linked sialic acid in hepatocellular carcinomas and preneoplastic hepatocellular lesions. Virchows Arch..

[122] Li Q., Wang F., Liu G., Yuan H., Chen T., Wang J., Xie F., Zhai R., Wang F., Guo Y. (2014). Impact of Mucin1 knockdown on the phenotypic characteristics of the human hepatocellular carcinoma cell line SMMC-7721. Oncol. Rep..

[123] Wang J., Liu G., Li Q., Wang F., Xie F., Zhai R., Guo Y., Chen T., Zhang N., Ni W. (2015). Mucin1 promotes the migration and invasion of hepatocellular carcinoma cells via JNK-mediated phosphorylation of Smad2 at the C-terminal and linker regions. Oncotarget.

[124] Lin Y.-S., Jung S.-M., Yeh C.-N., Chen Y.-C., Tsai F.-C., Shiu T.-F., Wu H.-H., Lin P.-J., Chu P.-H. (2009). MUC1, MUC2 and MUC5AC expression in hepatocellular carcinoma with cardiac metastasis. Mol. Med. Rep..

[125] Dai Y., Liu L., Zeng T., Liang J.-Z., Song Y., Chen K., Li Y., Chen L., Zhu Y.-H., Li J. (2018). Overexpression of MUC13, a Poor Prognostic Predictor, Promotes Cell Growth by Activating Wnt Signaling in Hepatocellular Carcinoma. Am. J. Pathol..

[126] Wang R., Chen L., Chen H., Hu L., Li L., Sun H., Jiang F., Zhao J., Liu G., Tang J. (2013). MUC15 Inhibits Dimerization of EGFR and PI3K–AKT Signaling and Is Associated With Aggressive Hepatocellular Carcinomas in Patients. Gastroenterology.

[127] Lin H., Yang B., Teng M. (2017). T-cell immunoglobulin mucin-3 as a potential inducer of the epithelial-mesenchymal transition in hepatocellular carcinoma. Oncol. Lett..

[128] Zhang H., Song Y., Yang H., Liu Z., Gao L., Liang X., Ma C. (2018). Tumor cell-intrinsic Tim-3 promotes liver cancer via NF-κB/IL-6/STAT3 axis. Oncogene.

[129] Liu F., Liu Y., Chen Z. (2018). Tim-3 expression and its role in hepatocellular carcinoma. J. Hematol. Oncol..

[130] Filmus J., Selleck S.B. (2001). Glypicans: Proteoglycans with a surprise. J. Clin. Investig..

[131] Hsu H.C., Cheng W., Lai P.L. (1997). Cloning and expression of a developmentally regulated transcript MXR7 in hepatocellular carcinoma: Biological significance and temporospatial distribution. Cancer Res..

[132] Zhu Z.W., Friess H., Wang L., Abou-Shady M., Zimmermann A., Lander A.D., Korc M., Kleeff J., Büchler M.W. (2001). Enhanced glypican-3 expression differentiates the majority of hepatocellular carcinomas from benign hepatic disorders. Gut.

[133] Capurro M., Wanless I.R., Sherman M., Deboer G., Shi W., Miyoshi E., Filmus J. (2003). Glypican-3: A novel serum and histochemical marker for hepatocellular carcinoma. Gastroenterology.

[134] Zhang J., Zhang M., Ma H., Song X., He L., Ye X., Li X. (2018). Overexpression of glypican-3 is a predictor of poor prognosis in hepatocellular carcinoma: An updated meta-analysis. Medicine.

[135] Capurro M.I., Xiang Y.-Y., Lobe C., Filmus J. (2005). Glypican-3 promotes the growth of hepatocellular carcinoma by stimulating canonical Wnt signaling. Cancer Res..

[136] Li L., Jin R., Zhang X., Lv F., Liu L., Liu D., Liu K., Li N., Chen D. (2012). Oncogenic activation of glypican-3 by c-Myc in human hepatocellular carcinoma. Hepatology.

[137] Zittermann S.I., Capurro M.I., Shi W., Filmus J. (2010). Soluble glypican 3 inhibits the growth of hepatocellular carcinoma in vitro and in vivo. Int. J. Cancer.

[138] Ruan J., Liu F., Chen X., Zhao P., Su N., Xie G., Chen J., Zheng D., Luo R. (2011). Inhibition of glypican-3 expression via RNA interference influences the growth and invasive ability of the MHCC97-H human hepatocellular carcinoma cell line. Int. J. Mol. Med..

[139] Wu Y., Liu H., Weng H., Zhang X., Li P., Fan C.-L., Li B., Dong P.-L., Li L., Dooley S. (2015). Glypican-3 promotes epithelial-mesenchymal transition of hepatocellular carcinoma cells through ERK signaling pathway. Int. J. Oncol..

[140] Ishiguro T., Sugimoto M., Kinoshita Y., Miyazaki Y., Nakano K., Tsunoda H., Sugo I., Ohizumi I., Aburatani H., Hamakubo T. (2008). Anti-glypican 3 antibody as a potential antitumor agent for human liver cancer. Cancer Res..

[141] Zhu A.X., Gold P.J., El-Khoueiry A.B., Abrams T.A., Morikawa H., Ohishi N., Ohtomo T., Philip P.A. (2013). First-in-man phase I study of GC33, a novel recombinant humanized antibody against glypican-3, in patients with advanced hepatocellular carcinoma. Clin. Cancer Res..

[142] Feng M., Gao W., Wang R., Chen W., Man Y.-G., Figg W.D., Wang X.W., Dimitrov D.S., Ho M. (2013). Therapeutically targeting glypican-3 via a conformation-specific single-domain antibody in hepatocellular carcinoma. Proc. Natl. Acad. Sci. USA.

[143] Ikeda M., Ohkawa S., Okusaka T., Mitsunaga S., Kobayashi S., Morizane C., Suzuki I., Yamamoto S., Furuse J. (2014). Japanese phase I study of GC33, a humanized antibody against glypican-3 for advanced hepatocellular carcinoma. Cancer Sci..

[144] Abou-Alfa G.K., Puig O., Daniele B., Kudo M., Merle P., Park J.-W., Ross P., Peron J.-M., Ebert O., Chan S. (2016). Randomized phase II placebo controlled study of codrituzumab in previously treated patients with advanced hepatocellular carcinoma. J. Hepatol..

[145] Gao W., Kim H., Feng M., Phung Y., Xavier C.P., Rubin J.S., Ho M. (2014). Inactivation of Wnt signaling by a human antibody that recognizes the heparan sulfate chains of glypican-3 for liver cancer therapy. Hepatology.

[146] Gao H., Li K., Tu H., Pan X., Jiang H., Shi B., Kong J., Wang H., Yang S., Gu J. (2014). Development of T cells redirected to glypican-3 for the treatment of hepatocellular carcinoma. Clin. Cancer Res..

[147] Zhai B., Shi D., Gao H., Qi X., Jiang H., Zhang Y., Chi J., Ruan H., Wang H., Ru Q.C. (2017). A phase I study of anti-GPC3 chimeric antigen receptor modified T cells (GPC3 CAR-T) in Chinese patients with refractory or relapsed GPC3+ hepatocellular carcinoma (r/r GPC3+ HCC). J. Clin. Oncol..

[148] Wu X., Luo H., Shi B., Di S., Sun R., Su J., Liu Y., Li H., Jiang H., Li Z. (2019). Combined Antitumor Effects of Sorafenib and GPC3-CAR T Cells in Mouse Models of Hepatocellular Carcinoma. Mol. Ther..

[149] Guo X., Jiang H., Shi B., Zhou M., Zhang H., Shi Z., Du G., Luo H., Wu X., Wang Y. (2018). Disruption of PD-1 Enhanced the Anti-tumor Activity of Chimeric Antigen Receptor T Cells Against Hepatocellular Carcinoma. Front. Pharmacol..

[150] Wice B.M., Gordon J.I. (1995). A tetraspan membrane glycoprotein produced in the human intestinal epithelium and liver that can regulate cell density-dependent proliferation. J. Biol. Chem..

[151] Li Y., Wang L., Qiu J., Da L., Tiollais P., Li Z., Zhao M. (2012). Human tetraspanin transmembrane 4 superfamily member 4 or intestinal and liver tetraspan membrane protein is overexpressed in hepatocellular carcinoma and accelerates tumor cell growth. Acta Biochim. Biophys. Sin..

[152] Kwon S., Choi K.-C., Kim Y.-E., Ha Y.-W., Kim D., Park B.K., Wu G., Kim D.-S., Lee Y., Kwon H.-J. (2014). Monoclonal antibody targeting of the cell surface molecule TM4SF5 inhibits the growth of hepatocellular carcinoma. Cancer Res..

[153] Zhao P., Zhang W., Wang S.-J., Yu X.-L., Tang J., Huang W., Li Y., Cui H.-Y., Guo Y.-S., Tavernier J. (2011). HAb18G/CD147 promotes cell motility by regulating annexin II-activated RhoA and Rac1 signaling pathways in hepatocellular carcinoma cells. Hepatology.

[154] Tang J., Guo Y.-S., Zhang Y., Yu X.-L., Li L., Huang W., Li Y., Chen B., Jiang J.-L., Chen Z.-N. (2012). CD147 induces UPR to inhibit apoptosis and chemosensitivity by increasing the transcription of Bip in hepatocellular carcinoma. Cell Death Differ..

[155] Wu J., Li Y., Dang Y.-Z., Gao H.-X., Jiang J.-L., Chen Z.-N. (2015). HAb18G/CD147 promotes radioresistance in hepatocellular carcinoma cells: A potential role for integrin β1 signaling. Mol. Cancer Ther..

[156] Wu B., Liu Z.-Y., Cui J., Yang X.-M., Jing L., Zhou Y., Chen Z.-N., Jiang J.-L. (2017). F-Box Protein FBXO22 Mediates Polyubiquitination and Degradation of CD147 to Reverse Cisplatin Resistance of Tumor Cells. Int. J. Mol. Sci..

[157] Peng F., Li H., You Q., Li H., Wu D., Jiang C., Deng G., Li Y., Li Y., Wu Y. (2017). CD147 as a Novel Prognostic Biomarker for Hepatocellular Carcinoma: A Meta-Analysis. Biomed. Res. Int..

[158] Cui J., Huang W., Wu B., Jin J., Jing L., Shi W.-P., Liu Z.-Y., Yuan L., Luo D., Li L. (2018). N-glycosylation by N-acetylglucosaminyltransferase V enhances the interaction of CD147/basigin with integrin β1 and promotes HCC metastasis. J. Pathol..

[159] Zhao S., Li H., Wang Q., Su C., Wang G., Song H., Zhao L., Luan Z., Su R. (2015). The role of c-Src in the invasion and metastasis of hepatocellular carcinoma cells induced by association of cell surface GRP78 with activated α2M. BMC Cancer.

[160] Luo C., Xiong H., Chen L., Liu X., Zou S., Guan J., Wang K. (2018). GRP78 Promotes Hepatocellular Carcinoma proliferation by increasing FAT10 expression through the NF-κB pathway. Exp. Cell Res..

[161] Zhang Y., Hu M.-Y., Wu W.-Z., Wang Z.-J., Zhou K., Zha X.-L., Liu K.-D. (2006). The membrane-cytoskeleton organizer ezrin is necessary for hepatocellular carcinoma cell growth and invasiveness. J. Cancer Res. Clin. Oncol..

[162] Kang Y.K., Hong S.W., Lee H., Kim W.H. (2010). Prognostic implications of ezrin expression in human hepatocellular carcinoma. Mol. Carcinog..

[163] Yeh C.-N., Pang S.-T., Chen T.-W., Wu R.-C., Weng W.-H., Chen M.-F. (2009). Expression of ezrin is associated with invasion and dedifferentiation of hepatitis B related hepatocellular carcinoma. BMC Cancer.

[164] Lu W., Yang C., Du P., Zhang J.-L., Zhang J.-C. (2017). Effects of arsenic trioxide on the expression of ezrin in hepatocellular carcinoma. Medicine.

